# Pressure Injuries in Medically Complex Children: A Review

**DOI:** 10.3390/children4040025

**Published:** 2017-04-07

**Authors:** Katherine Freundlich

**Affiliations:** Department of Pediatrics, Division of Hospital Medicine, Vanderbilt University Medical Center, Nashville, TN 37232, USA; katie.freundlich@vanderbilt.edu

**Keywords:** children with medical complexity, pressure injury, pressure ulcer

## Abstract

Pressure injuries are a challenging problem in the care of medically complex children. Available evidence is limited, and there are theoretical reasons to use caution before extrapolating adult data, including key differences in body composition, common locations of pressure injury, and association with medical devices. The focus of this article will be to review the definition of a pressure injury and what is known about pathophysiology, prevention, recognition, staging, and treatment of pressure injuries in children with medical complexity.

Pressure injuries are a challenging problem in the care of medically complex children. The National Pressure Ulcer Advisory Panel (NPUAP), an independent not-for-profit professional organization composed of members of different health care disciplines dedicated to the prevention and management of pressure injuries, defines pressure injuries as “localized damage to the skin and underlying soft tissue usually over a bony prominence or related to a medical or other device. The injury can present as intact skin or an open ulcer and may be painful. The injury occurs as a result of intense and/or prolonged pressure or pressure in combination with shear. The tolerance of soft tissue for pressure and shear may also be affected by microclimate, nutrition, perfusion, co-morbidities and condition of the soft tissue [1].”

Many factors place medically complex children at risk for these injuries. The National Institute for Health and Care Excellence (NICE), an independent organization established by the UK government to advise the National Health Service, published a guideline in 2014 for the prevention and management of pressure ulcers across all age ranges. Based on experience and opinion, the authors defined neonates, infants, children, and young people at risk for pressure ulcers as those who were admitted to secondary or tertiary care or those with a risk factor such as significant limited mobility, inability to reposition themselves, significant loss of sensation, nutritional deficiency, significant cognitive impairment, or previous or current pressure ulcer [2]. Definitions of children with medical complexity vary locally by program, but Cohen et al. put forward a useful framework to identify these children defined by the coexistence of four key features: multiple family-identified service needs, chronic conditions, functional limitations, and extraordinarily high health care use [3], features which overlap substantially with the pressure ulcer risk factors identified by the NICE. Review of the literature did not identify any studies that specifically identified the incidence or prevalence of pressure ulcers in medically complex patients. The point-prevalence of pressure ulcers of any stage in multi-center studies of pediatric patients in children’s hospitals ranges from 4% [4] to 35% [5], but these studies did not sufficiently characterize their populations to allow a clear estimate of the point-prevalence among pediatric patients with medical complexity.

Standard pressure injury risk assessment scales have been developed for pediatrics—the most common are the Braden Q, Garvin, and Glamorgan scales. In a performance comparison of these scales in children’s hospitals, the Glamorgan scale had the highest predictive ability [6] although comparison of the Braden Q and Glamorgan among pediatric inpatients has also concluded that either scale could be used [7]. Our literature review did not discover any risk assessment scales specifically validated for pediatric patients in the home setting although home health nursing literature has suggested that they could be used [8].

Research in pediatric pressure injury has lagged behind research in adult pressure injury. Before extrapolating adult data, however, practitioners caring for medically complex children should understand some differences between pressure injuries in children compared to those in adults. Body composition differs between pediatric and adult populations and changes rapidly with growth and development across the pediatric age range itself. In general, infants have less muscle and more fat than adults, which makes their subcutaneous tissue softer and more susceptible to deformation under the same force [9]. Skin characteristics also change with age in children. The slope of the stress–strain response in human skin, or in other words the amount of tension that occurs in response to a similar stress, starts out relatively high in early life, then decreases to a minimum between ages 15 to 25 [10]. Comorbidities in the medically complex population also impact body composition. Among children with cerebral palsy, those with greater functional impairment (Gross Motor Function Classification System Level III or IV) have higher fat percentages and lower lean body mass than children with less functional impairment (Gross Motor Function Classification System Level I or II) [11].

Body proportions also change as children grow. Through infancy and early childhood, the occiput is the largest bony prominence and a site of higher supine pressure than the sacrum—this changes for body surface areas above 1 m^2^ [12]. Although the sacrum, buttock, and heels are the most prevalent locations for pressure injuries in adults [13], the most common locations of pressure ulcers in pediatric patients in a tertiary care hospital were the ears and occiput [14].

Medical devices are an extremely important consideration as they are associated with 50% of pressure ulcers in pediatric inpatients [15,16]. In the pediatric intensive care unit (PICU) setting of a freestanding children’s hospital, devices most associated with pressure ulcers were non-invasive positive pressure facemasks, casts, and tracheostomy tubes and ties. Craniofacial anomalies were associated with 46% of the facemask pressure ulcers [16]. It is unclear how generalizable these findings may be to other settings. Although there have been less systematic studies in the home setting, it is important to note that many of these devices are used out-of-hospital in the medically complex population. Physical growth over time also theoretically puts patients at risk of outgrowing previously well-fitted devices.

As a result of the lag in pediatric-specific research, many management strategies have been adapted from adult literature and/or suggested based on expert opinion. Numerous strategies have been suggested to prevent pressure injuries. Repositioning more often than every four hours has been recommended for children at high risk of pressure injuries [2,16]. To reduce friction and shear with repositioning, a quality improvement bundle adopted in the intensive care unit (ICU) setting of one large, freestanding children’s hospital recommended using a draw sheet and repositioning devices for all patients. The bundle in its entirety resulted in a downward shift of mean pressure ulcer rate in the PICU [16].

The same bundle recommended using pressure reduction surfaces for chairs and adult beds, float heels, and “appropriate” seat cushions for chair-bound patients [16]. With regard to selecting a support surface, the 2014 NICE guidelines recommended using a high specification cot or bed mattress or overlay for pediatric patients with an existing pressure ulcer but did not make any specific recommendation for the support surface for the population at risk [2]. A high specification surface typically refers to a higher density or visco-elastic foam surface designed to redistribute pressure more effectively than a standard mattress [17]. Studies have examined interface pressure on various support surfaces for pediatric patients with differing results. A pilot study of healthy children under age six found that mean interface pressure with the occiput was lowest for air surface as compared to mattress, foam, fluidized, or gel materials [18]. Another study of healthy children of various ages found that interface pressure at the occiput was lowest with a Delta foam overlay alone or in combination with a Gel-E-Donut pillow as compared with a standard hospital mattress, Gel-E-Donut alone, or low air-loss bed [19]. Of note, some research has suggested using pressure reduction mattresses, which may have the unintended consequence of lower cardiopulmonary resuscitation (CPR) efficacy if performed on that surface although the use of a backboard can mitigate the negative effect [20]. With respect to wheelchair support surfaces, a small study of wheelchair-bound adult patients, mostly with cerebral palsy and scoliosis, showed that foam materials had lower interface pressures compared to gel materials [21]. We could not identify any studies of wheelchair seating materials specifically in wheelchair-bound pediatric patients in this review. Based on the available evidence discussed above, clinicians could reasonably select from several surfaces that provide superior protection to standard mattresses and may wish to use external factors such as cost, availability, and manufacturer specifications to determine a starting point.

A pediatric quality improvement initiative at a large freestanding children’s hospital included device-specific practice changes—specifically applying foam barrier dressing (Mepilex Transfer) to tracheostomy sites in the operating room at the time of initial tracheostomy placement and allowing a small air leak in pediatric Bilevel Positive Airway Pressure (BiPAP) masks. Respiratory device-related hospital-acquired pressure ulcers slightly decreased in frequency following adoption of the initiative [22]. A retrospective study comparing the rate of peristomal skin breakdown at first tracheostomy change before and after initiation of a standard practice of placing a silver-impregnated foam dressing (Mepilex Ag, Oakville, Ontario, Canada underneath new tracheostomy tubes and ties showed a decrease in skin break from 11.8% to 0% [23].

Proper care for the skin includes keeping it clean with avoidance of excess moisture or dryness. Specific measures adopted in the ICU quality improvement (QI) bundle described above included checking common moisture sites every 2–4 h, using diapers with a breathable outer cover, changing diapers as soon as wet, applying protective diaper cream, removing moisture from under devices, and keeping skin under casts, splints, braces, and collars clean and dry [16].

Providing optimal nutrition has also been recommended, specifically with respect to protein, hydration, caloric, and vitamin needs in growing children [24]. A case–control study of patients with and without pressure ulcers in a pediatric intensive care setting found that weight loss was a risk factor for pressure ulcers [25]. The ICU QI bundle above included an ongoing nutrition consult for all moderate- to high-risk patients [16]. We could not identify studies of any specific nutritional interventions with respect to their impact on pressure injuries in pediatric patients, so the definition of optimal nutrition remains somewhat unclear.

Measures to improve patient mobility and skin perfusion are also thought to promote pressure injury prevention, but these measures could vary based on the specific clinical situation.

Beyond prevention, the next important aspect of care is recognition of developing pressure injuries. Based on experience and opinion, the 2014 NICE guidelines recommend routine skin assessment by a trained healthcare professional for all children at high risk of pressure injury [2]. There is no clear evidence basis for how often a “routine” assessment should occur. A quality improvement bundle adopted in the ICU setting of one large, freestanding children’s hospital recommended daily head-to-toe evaluation of the entire body surface area and examination of the skin under each device every 12 h but acknowledged that this was time-consuming and difficult for skin under critical devices [16]. Compliance with daily head-to-toe evaluations averaged 81% in PICU and 50% in neonatal intensive care unit (NICU), and compliance with under-device assessments averaged 57% and 50% in the PICU and NICU, respectively [16]. An additional study would be needed to determine if the findings of compliance with skin evaluations are more widely generalizable. The feasibility of such assessments in the home setting is unclear although daily assessment of skin at bath time has been recommended [8]. Our review did not yield any studies of the ability of parents and/or home nurses to conduct accurate skin assessments. Cases of cutis aplasia and occult neural tube defects presenting as presumed pressure ulcers have been described in children, highlighting a unique differential diagnostic consideration for skin defects in the cranio-caudal axis in this population [26].

When recognized, injuries can be staged according to a standard framework as defined by the NPUAP:

- Stage 1—Non-blanchable erythema of intact skin

Intact skin with a localized area of non-blanchable erythema, which may appear differently in darkly pigmented skin. Presence of blanchable erythema or changes in sensation, temperature, or firmness may precede visual changes. Color changes do not include purple or maroon discoloration; these may indicate deep tissue pressure injury.

- Stage 2—Partial thickness skin loss with exposed dermis

Partial-thickness loss of skin with exposed dermis. The wound bed is viable, pink or red, moist, and may also present as an intact or ruptured serum-filled blister. Adipose (fat) is not visible and deeper tissues are not visible. Granulation tissue, slough, and eschar are not present. These injuries commonly result from adverse microclimate and shear in the skin over the pelvis and shear in the heel. This stage should not be used to describe moisture associated skin damage (MASD) including incontinence associated dermatitis (IAD), intertriginous dermatitis (ITD), medical adhesive related skin injury (MARSI), or traumatic wounds (skin tears, burns, abrasions).

- Stage 3—Full thickness skin loss

Full-thickness loss of skin, in which adipose (fat) is visible in the ulcer and granulation tissue and epibole (rolled wound edges) are often present. Slough and/or eschar may be visible. The depth of tissue damage varies by anatomical location; areas of significant adiposity can develop deep wounds. Undermining and tunneling may occur. Fascia, muscle, tendon, ligament, cartilage, and/or bone are not exposed. If slough or eschar obscures the extent of tissue loss, this is an unstageable pressure injury.

- Stage 4—Full thickness skin and tissue loss

Full-thickness skin and tissue loss with exposed or directly palpable fascia, muscle, tendon, ligament, cartilage, or bone in the ulcer. Slough and/or eschar may be visible. Epibole (rolled edges), undermining, and/or tunneling often occur. Depth varies by anatomical location. If slough or eschar obscures the extent of tissue loss this is an unstageable pressure injury.

- Unstageable—Obscured full thickness skin and tissue loss

Full-thickness skin and tissue loss in which the extent of tissue damage within the ulcer cannot be confirmed because it is obscured by slough or eschar. If slough or eschar is removed, a Stage 3 or Stage 4 pressure injury will be revealed. Stable eschar (i.e., dry, adherent, intact without erythema or fluctuance) on the heel or ischemic limb should not be softened or removed.

- Deep Tissue—Persistent non-blanchable deep red, maroon, or purple discoloration

Intact or non-intact skin with localized area of persistent non-blanchable deep red, maroon, purple discoloration or epidermal separation revealing a dark wound bed or blood filled blister. Pain and temperature change often precede skin color changes. Discoloration may appear differently in darkly pigmented skin. This injury results from intense and/or prolonged pressure and shear forces at the bone–muscle interface. The wound may evolve rapidly to reveal the actual extent of tissue injury or may resolve without tissue loss. If necrotic tissue, subcutaneous tissue, granulation tissue, fascia, muscle, or other underlying structures are visible, this indicates a full thickness pressure injury (Unstageable, Stage 3, or Stage 4). Do not use deep tissue pressure injury (DTPI) to describe vascular, traumatic, neuropathic, or dermatologic conditions [1].

Once pressure injuries are recognized, guidelines for prevention should be reviewed to avoid further tissue damage, and existing pressure injuries must be treated as well. Local wound care for open wounds (Stage 2 and above) includes debridement and wound dressing. Sterile water and normal saline have been recommended as cleansing agents for pediatric wounds [2]. Based on experience and opinion, the NICE guidelines recommend considering autolytic debridement with dressings for dead tissue and sharp and surgical debridement by trained staff if autolytic debridement is unsuccessful [2]. Autolytic debridement refers to using the body’s endogenous enzymes and moisture to re-hydrate, soften, and liquefy hard eschar and slough [27]. In practice, this may be accomplished by using a moisture-retentive dressing, such as a hydrogel, to soften covered by an absorptive, occlusive dressing, such as a polyurethane foam, to absorb the exudate that is produced; in some cases, this may be accomplished by using a dressing with some of both properties such as a hydrocolloid. A case study described successful use of medical honey to close a Stage 3 pressure ulcer on the leg of a premature infant, which that institution then adopted as a first-line therapy for Stage 2 and higher pressure ulcers replacing their prior practice of hydrogel plus secondary dressing; the case report did not provide any quantitative comparison of these two therapeutic approaches [28]. We could not identify a clear comparison of specific debridement approaches in children. Reasonable practice, however, would be to start with the least invasive and least painful approach, which is generally felt to be autolytic debridement, before moving on to other approaches if healing is not progressing.

The 2014 NICE guidelines recommend using dressings that promote a warm, moist environment for Grade 2, 3, and 4 pressure ulcers and recommend against gauze dressings based on low quality evidence [2]. There is very little evidence to guide decisions regarding which type of dressing to select; non-gauze dressing types that have been recommended for pediatric pressure ulcers include hydrocolloids, hydrogels, transparent films, and polyurethane foams [24]. In the absence of empiric evidence, a reasonable approach would be to choose specific dressings based on cost and availability within the general framework for autolytic debridement discussed above.

Adjunct therapies that have been proposed for pressure injury treatment include negative pressure wound therapy, hyperbaric oxygen therapy, ultrasound, and electrical stimulation. The 2014 NICE guidelines recommend not using hyperbaric oxygen or electrical stimulation therapy based on low quality evidence [2]. A recent Cochrane review of adult literature similarly found no strong evidence of benefit of electromagnetic therapy in treatment of pressure ulcers [29]. Based on low quality evidence and cost analysis, the NICE guidelines also do not recommend routine use of negative pressure wound therapy (more commonly called a wound vacuum) to treat a pressure ulcer [2] although this may be useful in some situations as suggested by a small case series of children with myelomeningocele-related pressure ulcers in which negative pressure wound therapy was used as a successful bridge to eventual skin graft and flap closure [30]. Even for adult patients, another recent Cochrane review found no randomized data regarding the role of reconstructive surgery in pressure ulcer management [31]. In practice, consultation with a plastic surgeon is typically considered in particularly severe cases of pressure injury or those that have worsened or failed to improve despite medical treatment.

Based on experience and opinion, the 2014 NICE guidelines recommend considering topical antimicrobial dressings where clinically indicated, such as in the context of spreading cellulitis, as well as systemic antibiotics for clinical evidence of local or systemic infection in neonates, infants, children, and young people [2]. These guidelines do not recommend routine use of antiseptics or antimicrobials to treat a pressure ulcer based on low quality evidence [2]. In another recent Cochrane review of studies in adults, where differences in wound healing were found between topical antimicrobial and other therapies, comparator treatment without antimicrobial properties was sometimes favored although overall evidence quality was low [32].

Pain control has also been recommended as an important adjunct therapy. Combined with maintaining the patient’s dignity, pain control may be the primary driver for therapy selection when a family’s goals are comfort-focused [24].

In conclusion, clinicians caring for medically complex children must be guided by available evidence, albeit imperfect, in combination with clinical reasoning in selecting the best prevention, recognition, and treatment strategies for pressure injuries in these patients. Certainly, available literature suggests that attention must be paid to the specific issues of body composition and growth in this population as well as the impact of a multitude of medical devices. Available pediatric literature is largely focused on the inpatient environment, so future study is especially needed in recognition and management of pressure injuries in medically complex children in the outpatient setting.
